# The Maternal Support Framework Studying Mothers’ Perceived Understanding and Support During Excessive Infant Crying: Exploratory Qualitative Study

**DOI:** 10.2196/75669

**Published:** 2025-10-28

**Authors:** Oona Janssens, Anna Galle, Ineke De Kruijff, Katrijn Brenning

**Affiliations:** 1 Department of Public Health and Primary Care Faculty of Medicine and Health Sciences Ghent University Ghent Belgium; 2 Department of Pediatrics St Antonius Ziekenhuis Utrecht The Netherlands; 3 Department of Developmental, Personality and Social Psychology Faculty of Psychology and Educational Sciences Ghent University Ghent Belgium

**Keywords:** excessive crying, infant, maternal support, parental distress, framework, maternal support framework

## Abstract

**Background:**

Excessive infant crying affects approximately 20% of families and can lead to parental distress, anxiety, and strained relationships. Despite its prevalence, many parents report feeling misunderstood and unsupported during these challenging periods.

**Objective:**

This study aimed to gain in-depth insight into mothers’ perceptions of being understood and supported in the context of excessive infant crying, focusing on three key stakeholder groups: partners, the personal network, and health care professionals. Based on these results, the study sought to develop a maternal support framework regarding excessive crying that could guide future research and practice, as well as support strategies.

**Methods:**

Using a qualitative approach supplemented by quantitative measures, through an online survey, three open-ended questions were included on how mothers would like to be understood and supported by the three stakeholder groups (ideal situation) and six 6-point Likert scales on mothers’ current perceived understanding and support regarding the three stakeholder groups (current situation). Descriptive statistics were used to examine current levels of understanding and support, and an inductive thematic analysis was applied to identify the ideal key support elements.

**Results:**

Data were collected from 432 mothers (n=238, 55.1% Dutch; n=194, 44.9% Flemish; mean age 33 years, range 21-45 years). Regarding the current situation, mothers rated health care professionals lowest in perceived understanding and support, with 50.6% (n=219) feeling little or no understanding and 47.1% (n=203) reporting little or no support. Similar patterns were found in the personal network (n=184, 42.6%, and n=164, 38%, respectively). Partners were perceived as most supportive, with only 17.6% (n=76) of mothers reporting little or no understanding and 21.8% (n=94) reporting little or no support. Based on the thematic analysis of the qualitative data, the ideal situation was framed in the newly developed maternal support framework. This framework identifies 25 distinct support forms, of which 12 (48%) are common support forms (partner, personal network, health care professionals, eg, listen actively), 6 (24%) are related to partners (eg, be physically present), 6 (24%) concern the support of health care professionals (eg, refer appropriately), and 1 (4%) is specific to the personal network (cope with the crying).

**Conclusions:**

This study underscores the need for holistic, family-centered approaches to supporting families with excessively crying infants. The proposed maternal support framework offers a foundation for developing tailored interventions that reflect the diverse roles of partners, personal networks, and health care professionals in maternal well-being.

## Introduction

Infant crying is an essential aspect of early development, serving as the primary mode of communication during the first months of life [1,2]. Although crying is typical behavior, it becomes a concern when approximately 20% of infants exhibit excessive crying [3]. Traditionally, excessive crying has been defined using objective criteria, such as Wessel’s rule of threes (crying for at least 2 hours per day, 3 days per week, for 3 consecutive weeks [4]), and, more recently, the ROME-IV criteria (recurrent, unexplained crying or irritability in infants under 5 months old that cannot be soothed by caregivers [5]). Although ROME-IV incorporates caregiver reports, its focus remains on observable infant behavior and colic specifically, offering limited attention to parental distress or the broader psychosocial context. Recent research emphasizes the limitations of such symptom-based classifications, highlighting the need to consider parents’ subjective experiences, such as stress and helplessness [6,7]. Indeed, Reijneveld et al [7] emphasize the importance of definitions that integrate both crying duration and the distress experienced by parents. Additionally, some researchers distinguish between “excessive crying,” which is based on parents’ subjective perception that their baby cries too much and signals a potential problem, and “prolonged crying,” which refers to a measurable duration [8]. This dual perspective highlights the growing recognition of parental perceptions and the intricate interplay between infant behavior and parental mental health. This is a positive development, as it underscores the importance of adopting a biopsychosocial perspective and approach to excessive crying [9], particularly given that a medical cause is identified in fewer than 5% of cases [1].

The experience of having an excessively crying infant can profoundly affect parents, leading to heightened levels of distress, anxiety, and depression, as well as difficulties bonding with their child [3,6,10-12]. These psychological challenges are often compounded by practical issues, such as early cessation of breast feeding, overfeeding, and, in some cases, long-term developmental risks for the infant. Although more research is still needed on the long-term consequences of excessive crying, excessively crying infants seem to be at increased risk of developing psychological, behavioral, and allergic disorders. In addition, there might be a higher incidence of headache and migraine later in life in children who cry excessively [13]. In severe instances, excessive crying might also increase the risk of shaken infant syndrome [9-11,14]. These far-reaching effects underline the necessity of addressing excessive crying, not merely as an infant issue, but also as a family-centered concern that prioritizes the well-being of parents [6].

A qualitative study by Garratt et al [6] explored UK parents’ experiences of having an excessively crying baby. Their study showed that parents often have disrupted expectations and experiences of parenthood, perceive stigma and social isolation, and seek support and validation of their experience [6]. This sense of isolation is further amplified by dissatisfaction with health care; research shows that 55% of the participating Dutch mothers in a study by van der Veek et al [15] felt that they received inadequate information about their infant’s crying and available health care support. Therefore, proactively providing comprehensive information during pregnancy or shortly after birth might better equip parents to manage the challenges associated with excessive crying [6]. In the same vein, Harskamp et al [16] explored the types and format of health care support parents with excessively crying infants need. They identified four key themes: (1) the need for confidence in health care professionals, (2) the need to investigate potential somatic causes of crying, (3) the need for acknowledgment of their baby’s crying, and (4) the need for support to break the cycle of exhaustion and self-doubt [16]. These findings underscore the importance of holistic, family-centered care that addresses not only the crying itself but also the emotional and practical challenges faced by parents. Providing tailored support is essential for preventing cascading difficulties and fostering positive parent-infant interactions [17].

Even though health care professionals are thus critical in providing support and care for parents with an excessively crying baby, an integrative approach involving the entire support system of parents with an excessively crying baby is essential. Moreover, existing research often isolates specific aspects of excessive crying, such as health care interventions, for example, manual therapy [18,19], probiotics [20], parental coping [6], or soothing techniques (eg, swaddling [21]). Furthermore, studies on excessive crying are often small in scale, and an integrative care framework for parents with excessively crying infants is lacking.

To optimally identify the needs of parents with excessively crying infants, it is crucial to assess both their current experiences and their perceptions of an ideal support situation. Previous research has shown that mothers often feel they are not taken seriously or that the severity of their situation is not adequately understood or acknowledged [15]. Additionally, receiving insufficient or inadequate support has been highlighted as a key issue by van der Veek et al [15]. Importantly, being understood and receiving support represent two distinct but interrelated needs for families facing excessive infant crying. Building on this, this survey study investigated mothers’ perceived understanding and support from their partners, personal networks, and health care professionals (current situation). Moreover, building on the needs for support expressed by mothers (ideal situation), this study aimed to develop a comprehensive conceptual framework—the maternal support framework—that can serve as a foundation for future research and interventions designed to support families with excessively crying babies.

## Methods

### Sample

Recruitment began in January 2024. Using a cluster sampling technique, invitations to participate were distributed via specific Facebook groups for parents, such as “Mama’s,” “Mama’s onder elkaar,” and “Onder mama’s.” Additionally, invitations were also shared in more specialized groups focusing on excessively crying infants, such as “Reflux Baby,” “Crying Baby Parents,” “Crying Baby Support Center,” and similar communities. A link was available to participate in the study. The relatively homogeneous sample, including only mothers who had experienced having a crying baby themselves (without a specific quantification of the crying amount, as our main focus was on parental perception) allowed for an in-depth exploration of the phenomenon of excessive crying and, more specifically, parents’ perceptions of understanding and support during this life phase. Although there is no gold standard for determining the sample size in qualitative research, we aimed to recruit and collect data until we reached data saturation. However, 432 participants completed the survey within a week, which could be seen as an exceptional response rate, so we decided to include all data, thereby further strengthening the validity of the study.

### Ethical Considerations

When interested mothers wanted to participate, they could click an anonymous link that directed them to a questionnaire. The study received approval from the Ethics Committee of Ghent University (reference number: 2023-141W). Prior to commencement of the questionnaire survey, written informed consent was obtained from all participants after providing them with comprehensive information about the study goals, study flow, etc. Additionally, participants acknowledged a privacy statement at the beginning of the questionnaire, which provided comprehensive information regarding General Data Protection Regulation (GDPR) compliance and the handling of their data. No incentives were provided. A brief overview of the sample can be found in the *Results* section.

### Data Collection

A questionnaire was developed in Qualtrics. The questionnaire was based on previous research indicating a gap in knowledge about the perceived understanding and support of mothers’ direct environment (partners, personal networks, and health care professionals). First, the invitation was posted in the aforementioned Facebook groups to test the questionnaire and ensure the questions are clear. Three mothers pilot-tested the questionnaire using a think-aloud procedure where they could write down their concerns regarding the questions. However, no ambiguities was identified, as expected, as the questions were straightforward and concise. Therefore, the final questionnaire consisted of 11 questions that were a combination of qualitative and quantitative measures. Nine items concerned perceptions of the current (six items) and the ideal (three items) situation, one item was an open-ended question on which health care professionals they consulted during this period, and one item was on the general daily duration of crying. Participants were included based on maternal characteristics—specifically, having experienced a baby who cried excessively at the time of the study or in the past.

#### Qualitative Measures

To encourage deep insights and strengthen our qualitative research approach by data triangulation, a combination of open-ended questions and Likert scales was used. The qualitative part existed of asking participants how they wanted to be understood and supported by each of the groups (partners, personal networks, and health care professionals in general=ideal situation). Finally, they were asked which health care professionals they consulted during this period.

#### Quantitative Measures

In addition to answering the open-ended questions, participants scored six Likert scales that assessed their perceptions (current situation) of understanding (three questions) and support (three questions) from their (1) partners, (2) personal networks (parents, grandparents, friends, colleagues, etc), and (3) consulted health care professionals (health care disciplines not specified). These questions used a 6-point Likert scale with the following verbal anchors: perfect (1), very good (2), good (3), fair (4), poor (5), and none (6). The use of these verbal anchors avoids numeric values and clearly defines the scale in verbal terms. We chose 6 points to prevent consistently selecting the neutral midpoint [22-25]. Finally, a scale with predefined time categories (in hours) was used to assess the duration of crying in different parts of the day as part of the demographic data. The complete questionnaire can be found in Multimedia Appendix 1.

### Data Analysis

This study used a qualitative research approach (open-ended questions) supplemented by quantitative measures (Likert scales).

#### Qualitative Measures

Open-ended questions that explored how participants wished to be supported and understood were analyzed using an inductive content analysis approach in NVivo [26]. Given the exploratory nature of the study and the straightforward structure of the data, a single experienced coder conducted the initial coding. Thereby, the data were first thoroughly read multiple times to gain a deep understanding of the content. Following this, the organizing process began, which included open coding, theme development, and abstraction [27]. The final phase of abstraction involved creating overarching concepts. Afterward, an independent reviewer examined the themes to eliminate ambiguity, overlap, and potential misinterpretations, thereby enhancing credibility and reducing potential bias. This approach ensured a balance between analytical consistency and critical validation. Additionally, the frequency with which each theme emerged in the data was calculated to assess how well the integration of themes resonated with participants. This approach allowed for an estimation of the importance and prevalence of the identified themes, ultimately contributing to the development of a framework for the overarching concept of “care for parents with an excessively crying baby,” referred to as the maternal support framework [28].

#### Quantitative Measures

Analysis of the Likert scales involved descriptive statistics to calculate frequencies, as well as minimum, maximum, and mean values.

## Results

### Participant Details

A total of 432 mothers fully completed the questionnaire. Of these, 55.1% (n=238) resided in the Netherlands, while 44.9% (n=194) were from Flanders (Belgium). Additionally, 0.5% (n=2) of the participants reported living in other countries, specifically France or Italy. The average age of the participants was 33 years (range 21-45). The number of children per participant varied from 1 to 5, with a mean of 1.77 children per parent.

Descriptive analyses showed that the 432 participating mothers consulted a total of 1111 different health care professionals at least once during the period their baby cried excessively. On average, each mother consulted 2.7 health care professionals due to their infant’s excessive crying. These health care professionals included pediatricians, general practitioners, midwives, child health nurses, osteopaths, pharmacists, physiotherapists, psychologists, chiropractors, dentists, radiologists, parenting coaches, lactation consultants, surgeons, dietitians, and occupational therapists. The most frequently consulted health care professionals were pediatricians (305/408, 74.8%, mothers), general practitioners (212/408, 52%, mothers), child health nurses (153/408, 37.5%, mothers), osteopaths (121/408, 29.7%, mothers), and midwives (112/408, 27.5%, mothers). Table 1 provides a detailed overview of consulted health care professionals and the number of mothers who consulted them.

Excessive crying, on average, began approximately 2 weeks and 1 day after birth. Parents reported that they typically noticed their baby’s crying was above average around 4 weeks and 2 days after birth. Participants estimated that their babies cried for nearly 12 hours (mean 11.97 hours, SD 5.2) each day, with the most significant crying occurring in the evening, averaging 6.6 hours (SD 5.3). This was followed by crying in the afternoon, which averaged 5.5 hours (SD 5), while mornings saw the least crying, averaging about 4.4 hours (SD 4.7).

**Table 1:** Overview of consulted health care professionals (N=1111).

Health care professionals	Mothers (N=432), n (%)
Missing (participant did not specify consulted health care professionals)	24 (5.6)
None	1 (0.2)
Pediatrician	305 (70.6)
General practitioner	212 (49.1)
Child health nurse: *Kind en Gezin* (Belgium) or *Consultatiebureau* (the Netherlands)	153 (35.4)
Osteopath	121 (28.0)
Midwife	112 (25.9)
Child physiotherapist	31 (7.2)
Other alternative medicine practitioner (eg, homeopath, acupuncturist)	27 (6.3)
Emergency doctor	23 (5.3)
Lactation consultant	22 (5.1)
Psychologist	22 (5.1)
Chiropractor	12 (2.8)
Dietitian	9 (2.1)
Manual therapist	6 (1.4)
Allergist	6 (1.4)
Baby therapist	5 (1.2)
Sleep coach	5 (1.2)
Manual therapist	5 (1.2)
Nurse	5 (1.2)
Foot reflexologist	5 (1.2)
Speech therapist	5 (1.2)
Dermatologist	3 (0.7)
Ear, nose, and throat specialist	3 (0.7)
Mesologist	3 (0.7)
Neurologist	2 (0.5)
Dentist	2 (0.5)
Psychiatrist	2 (0.5)
Pedagogue	2 (0.5)
Neonatologist	1 (0.2)
Orthomolecular therapist	1 (0.2)

### Current Situation of Understanding and Support

**Figure 1:**
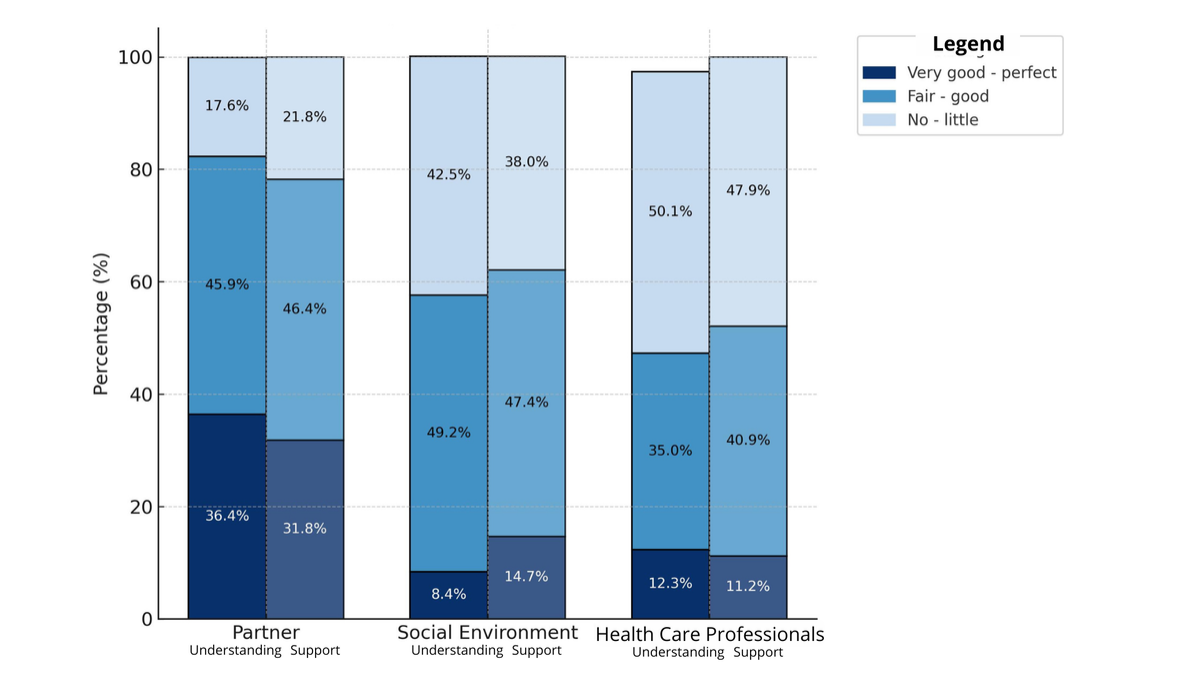
Visualization of mothers' perceptions.

Based on the quantitative measures, mothers’ perception of understanding and support from *health care professionals* was the lowest, with a score of 4.4 and 4.3, respectively, on a 6-point Likert scale (ranging from 1=perfect understanding/support to 6=no understanding/support). Half of the parents (n=216, 50%) felt they perceived no to little understanding from their health care professionals. The same was seen for perceived support, where 47% (n=203) of the parents felt they perceived little to no support. However, it is important to note that no differentiation was made among the health care professionals involved. The results therefore offer a general overview of health care professionals as a whole. Although parents’ perceptions of understanding and support from health care professionals were the lowest, there is also room for improvement in the understanding and support from parents’ partners and personal networks. A large group of parents reported receiving little to no understanding (n=76, 17.6%; mean=3.1/6) and support (n=94, 21.8%; mean score=3.3/6) from their *partners*, as well as little to no understanding (n=184, 42.6%; mean score=4.2/6) and support (n=164, 38%; mean score=4/6) from their *personal networks*. An overview can be found in Figure 1.

### Ideal Situation of Understanding and Support

Of the 432 participants, 423 (97.2%) responded to the open-ended questions about how they want understanding and support from their partners, personal networks, and health care professionals to be improved. It was striking that when people were asked to name their needs, they tended to focus not on their active needs but rather on negative experiences in which their needs had not been met. These narratives shed light on the unmet expectations and frustrations of mothers navigating the challenges of excessive infant crying. For example, one mother shared:

I wish certain people would have acknowledged it. And that they wouldn’t accuse me of keeping my child away from family while I was acting in my child’s best interest, and they were unwilling to adapt.

The Netherlands, 29 years old, consulted a general practitioner, a child health nurse, a pediatrician, and a dermatologist

Another mother noted:

They dismissed me as a young mom, ignorant, first child, and all that.

Belgium, 26 years old, consulted several pediatricians and general practitioners

**Figure 2:**
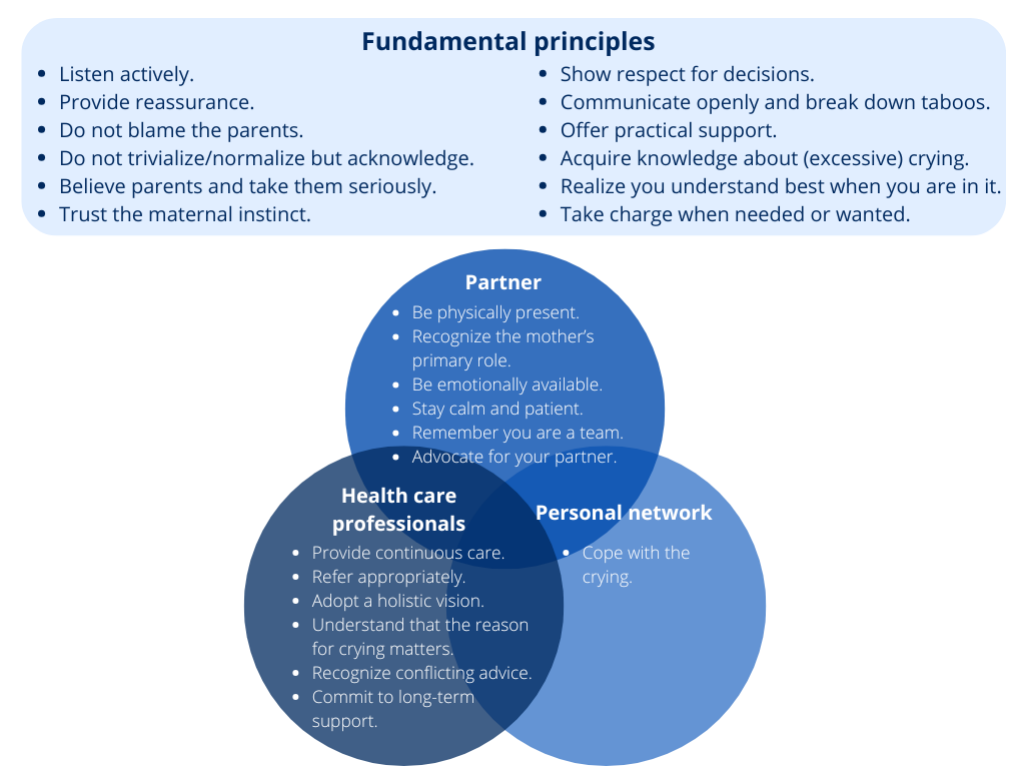
Maternal support framework.

These experiences, although painful, were crucial in identifying the gaps in support and helped shape the themes and subthemes, after which the overarching themes were transformed to support forms. Overall, the findings revealed that although some forms of support were shared across groups and incorporated as fundamental principles, others were more specific to the roles of partners, personal networks, or health care professionals. These forms of support were integrated through an in-depth analysis into the maternal support framework presented in Figure 2.

A total of 25 support forms or themes were identified, of which 12 (48%) were common across all three groups and were therefore labeled as *fundamental principles*, such as “communicate openly and break down taboos.” A participant’s quote below emphasizes the taboo around admitting struggles during early parenthood while she points out a lack of support options:

It’s a shame that there is still so much taboo around this, and that having a baby is still expected to be mostly “joyful,” while in reality, when you truly talk to people, you realize that many parents struggle and that for a lot of them, their “cloud” is more gray than pink. It’s also frustrating that society expects you to be back at work three months after giving birth, functioning just as before—whether you have a high-needs baby, sleep deprivation, or not. Additionally, there are very few support options or places where you can seek help for yourself and your baby. You can talk to a psychologist, but even there, waiting lists are always an issue.

Belgium, 35 years old, consulted a midwife, a general practitioner, and a psychologist

Another lamented the lack of acknowledgment of their struggles:

Since the baby was growing well, there was no problem. My own struggles were not acknowledged.

Belgium, 38 years old, consulted a pediatrician

Although many support forms were shared across groups (fundamental principles), some were specific to particular groups. For example, mothers expressed distinct expectations from their *partners*, such as “stay calm and patient” and “remember you are a team.” This resulted in the development of six specific partner support forms. One mother recounted:

My partner had no patience with our crying baby. It frustrated him a lot, and he took it out on me. It would have been really helpful if he could have stepped in with patience and acceptance sometimes.

The Netherlands, 29 years old, consulted a child health clinic

Another mother highlighted the value of teamwork, stating:

My partner and I have really been a team during this challenging time. He has pulled me through, and I have done the same for him.

The Netherlands, 27 years old, consulted a child health clinic, a general practitioner, and a pediatrician

These accounts underscore the critical role of partners in alleviating the mental and emotional burden of excessive infant crying.

Similarly, the support forms directed at *health care professionals* were particularly distinct, with six support forms specific to this group, for example, “provide continuous care” and “refer appropriately.” One mother expressed frustration, saying:

It is important that these parents feel heard. Don’t let them struggle on their own. I only realized later that my baby was a high-needs baby, even though I had asked the consultation clinic for help so many times. I felt so alone. It turned out that our hospital had a special clinic for crying babies—they probably could have helped much sooner. This might have spared me from depression.

The Netherlands, 37 years old, consulted a general practitioner and a pediatrician “too late”

These examples highlight the need for health care professionals to go beyond surface-level assessments and consider both parents’ and infants’ physical and emotional well-being.

The *personal network*, although less distinctive and showing more overlap with the other support forms, is also a critical source of either comfort or frustration. The most important theme related to the personal network that distinguished itself from the other two groups was to cope with crying. This was illustrated, for example, in the following quote:

When they came to visit and saw that he wouldn’t stop crying, they often couldn’t leave fast enough.

Belgium, 38 years old, consulted a midwife, a pediatrician, and a general practitioner

However, concerning their personal networks, mothers also called for several fundamental principles such as “offer practical support.” For example, one mother shared:

Avoid giving generic advice—just listening is enough. Offer to watch the baby for a few hours so they can recharge, take over household chores, or prepare dinner.

Belgium, 28 years old, consulted a general practitioner, a pediatrician, an osteopath, a homeopath, and a physiotherapist

## Discussion

### Principal Findings

This study, using a qualitative approach supplemented with quantitative measures, explored maternal perceptions of understanding and support when faced with an excessively crying baby (current situation), as well as the key forms of support the mothers would like to receive (ideal situation). With 432 participants, it offered a robust and diverse dataset, enabling nuanced insights into support needs and identifying potential forms of support from partners, personal networks, and health care professionals. These findings informed the development of the maternal support framework.

#### Crying Duration

The reported average of nearly 12 hours of infant crying per day appears unusually high compared to existing epidemiological data, even for excessively crying infants. This likely reflects a combination of recall bias, parental misinterpretation of crying versus fussing or general wakefulness, and the low coping ability of parents. Parent-reported measures, although practical for large-scale studies, are known to diverge from objective recordings. The Barr’s Baby Diary validation study found moderate correlations between diary reports and audio recordings for crying (r=0.67) but low or no correlation for fussing (r=0.45 for crying+fussing; r=0.01 for fussing alone [29]). As Vermillet et al [6] noted, this may be due to parents’ sensitivity to visual signs of distress not captured by audio, as well as the more fragmented nature of fussing behavior. As capturing the objective crying duration of these infants was not the main purpose of this study, these findings reflect perceived, not actual, cry durations, yet they remain valuable for understanding parental experience, which plays a crucial role in early caregiving and stress [2].

### Consulted Health Care Professionals

Before turning to the study’s central research questions on current and desired support, we highlight one key finding regarding health care use among mothers of excessively crying infants. The level of health care use, reflected in an average of 2.7 health care professionals consulted during periods of excessive crying, was relatively high in this study and aligns with previous research [15]. One reason for this is that many mothers feel a strong need to find an explanation for their baby’s excessive crying. However, in 95% of cases, no medical cause is found [1,30], and crying often decreases by the time the baby is 3-4 months old [31,32]. This suggests that the reduction in crying may be due to other factors, not just the health care professionals parents consult, and “finding the cause” of excessive crying is not the primary reason for its reduction [1,33,34]. For example, the months wherein parents consult different health care professionals might cover the natural peak of infant crying [2]. Consulting various health care professionals likely gives parents a sense of control, as they hope for a quick fix, which for them often means receiving a medical label [35]. However, this high health care consumption in combination with the low satisfaction of parents regarding health care delivery also highlights a lack of continuous and solid support, leaving parents feeling that they must take charge in the search for a cause, since they seem to believe that without a medical cause, there is no solution to excessive crying.

As for the specific health care professionals consulted, mothers most frequently sought help from a pediatrician, a general practitioner, child health nurses, an osteopath, or a midwife. Remarkably, no mother mentioned consulting an infant mental health (IMH) specialist—a health care professional with additional training, skills, and experience in working intensively with young children and families through a comprehensive, relationship-based approach [36]. One possible explanation is that parents, given the recent development of this field, are not yet aware of the existence of these specialized professionals. Another possibility is that parents are aware of a professional’s IMH training but simply do not specify it. Lastly, parents may have consulted such specialists without realizing their additional expertise. However, future research could help demonstrate the added value of such specialists in cases of excessive crying, as current research lacks a clear link to this issue, even though the existence and role of these specialists are increasingly being acknowledged [37].

#### Current and Ideal Situations of Understanding and Support

Mothers of excessively crying babies reported the lowest levels of understanding and support from health care professionals, with half feeling they received little to no understanding and support. These numbers are in accordance with previous research indicating that 55% of mothers feel they receive inadequate information regarding their baby’s crying and their caregiving role [15]. Although health care professionals showed the greatest need for improvement, mothers also experienced significant gaps in support and understanding from their partners and personal networks. This suggests widespread challenges in securing sufficient support networks for mothers.

This brings us to the qualitative data, which provided deeper insight into mothers’ perceived support needs. An in-depth analysis of the data led to the development of a maternal support framework consisting of 25 maternal support forms divided into common maternal support forms or fundamental principles and specific maternal support forms. Twelve general maternal support forms were identified, which applied to each of the groups (partners, personal networks, and health care professionals). Six maternal support forms were only detected toward health care professionals, six were identified toward mothers’ partners, and one was specified for their personal networks.

First, *common maternal support forms* were identified. The support forms identified in this qualitative study generally align with various needs previously reported in the literature. For example, the theme *communicate openly and break down taboos* corresponds to the stigma surrounding excessive crying, often exacerbated by societal taboos, which leaves mothers feeling isolated and unsupported. Clearly, insufficient social recognition and a lack of open communication can significantly increase maternal distress [6,7]. Moreover, prior research states that excessive crying often disrupts parental identity and generates stigma [6]. This highlights the critical need to explicitly address the taboo and blame surrounding infant crying in intervention programs, as doing so may reduce stigma, foster parental acceptance, and encourage seeking support. Furthermore, *practical support,* such as household tasks or caregiving assistance, was another recurrent theme. Many mothers reported that small gestures, such as offering to babysit or prepare a meal, could have alleviated their overwhelming burden. This resonates with studies advocating for community-based interventions to enhance family resilience during challenging periods [16].

Second, the findings regarding current and ideal levels of understanding and support by *health care professionals* highlight critical areas for improving health care delivery. Indeed, mothers expressed dissatisfaction with fragmented or dismissive health care, where excessive crying was trivialized or attributed to “normal” infant behavior without thorough investigation. Despite frequent consultations, health care professionals seem to underperform in meeting parental needs during episodes of excessive infant crying. This may be due to a predominant biomedical approach focused on ruling out disease or normaliz*ing* the crying behavior, rather than addressing the emotional and informational needs of parents [15,16]. Harskamp-Van Ginkel et al [16] stated that through the development of eight consecutive steps in the assessment of an excessively crying infant, *taking parents seriously* while demonstrating medical expertise and *offering a practical plan* is the way to go. However, *providing care continuity* with *appropriate referral* to health care professionals who can offer the most appropriate care at the time of the request for help and a *holistic vision* of both the baby’s and the mother’s well-being can improve mothers’ satisfaction and families’ well-being, as also shown in previous research [38]. The study underscores the need for a holistic screening approach that goes beyond quantifying crying episodes. Incorporating emotional and contextual factors into assessments could facilitate targeted interventions and foster greater understanding among health care professionals. Such approaches would align with calls in the literature for more family-centered care models that prioritize both infant well-being and parental mental health [7,39]. A great example of such a good practice, where a multidisciplinary approach is used with a focus on continuity of care, is the care model described by de Graaf et al [40], which is currently being further developed at the Sint-Antonius Hospital in the Netherlands. This integrated model supports families with excessively crying infants by combining psychosocial support, timely medical consultation, and structured follow-up across disciplines. It involves early intervention through clear guidance on soothing techniques and access to reliable (digital) information about excessive crying for all parents. In cases where a medical cause is suspected, parents are offered a joint consultation with both a pediatrician and a medical pedagogical professional within 5 working days. This close collaboration ensures that both medical and psychosocial aspects are addressed simultaneously. To safeguard continuity of care, a follow-up handover takes place within 2 weeks via a video call in the presence of the parents, allowing the referring professional to remain involved and ensuring a warm and informed transfer of care [40]. Accordingly, the recently validated Infant Crying and Parent Well-Being (ICPW) screening tool could serve as a starting point for a more nuanced approach to identify parents whom have a problem with the excessive crying of their infant. This ICPW screening tool is a brief, five-item instrument designed to identify parents who are struggling with their infant’s crying (example item: “Has your baby’s crying or fussing been a problem or upsetting to you recently?” Yes/No). It quickly and efficiently identifies families that are in need of extra support in comforting their child, considering parent-specific stressors, such as parenting stress. Its simplicity and clinical relevance make it well-suited for routine use in early infancy to guide timely support and referrals [41,42].

Third, the findings shed light on the forms of support that mothers consider important to receive from their *partners*. Participants highlighted the importance of a unified approach, where *partners act as advocates* *and teammates* in managing the situation. The strain of excessive crying often amplified the mental load for mothers, particularly when partners were perceived as less *present, engaged*, or understanding. Conversely, where strong partner support was present, mothers reported greater emotional stability and a sense of shared responsibility. These findings support the literature emphasizing the protective effects of strong partner relationships in mitigating stress [39]. However, having an excessively crying baby might cause marital stress, arguments, and feelings of resentment toward the partner, which confirms our findings [39].

Fourth, the forms of maternal support from the *personal network* were explored. Mothers often felt that their networks failed to grasp the intensity of their experiences, leading to feelings of alienation. *Experiential knowledge* where social contacts had faced similar challenges was perceived as enhancing understanding and empathy. This suggests a potential benefit of peer support programs or initiatives that connect parents with others who have navigated similar challenges [43]. Given that mothers indicated their personal networks often had difficulty *coping with their baby’s crying*, and considering the lack of research on this topic, increasing awareness and knowledge, as well as conducting further investigation, may be warranted.

The results of this study also highlight gaps in research and practice. Existing studies often focus on isolated elements of the parent’s environment, such as partner support or health care delivery, while neglecting the broader support system. This fragmented nature of current research also limits generalizability. The findings of this study provide a first step in expanding the scope of research to encompass a fuller spectrum of maternal experiences. However, including the role of societal pressure, for instance, could yield valuable insights into developing comprehensive support systems. In addition, the results emphasize the importance of *fostering greater knowledge* and understanding of excessive crying among different central stakeholders—parents, personal networks, and health care professionals. Enhancing awareness could *reduce stigma*, *improve acknowledgment* of parental struggles, and ultimately lead to better care and support. Such efforts could transform excessive crying from an isolating and distressing experience to an opportunity for building stronger family and community resilience. Finally, the findings of this study underscore the need for a *holistic and context-sensitive approach* to understanding and addressing excessive crying, both in research and in practice.

### Practical Implications

The maternal support framework may serve as a valuable foundation for the development of new support programs or the optimization of existing ones. By offering a clear representation of the support needs of the target population, the framework can enable health care professionals and program developers to assess which needs are already being addressed and to identify how to broaden their interventions in line with unmet parental support needs. For example, the framework’s emphasis on fundamental principles, such as “listen actively,” “believe parents and take them seriously,” and “provide reassurance,” shows the need for interventions to create safe spaces where parents can share their feelings without judgment, reducing the taboo and blame associated with infant crying; the framework’s emphasis on trusting the maternal instinct could help develop family-centered interventions focusing on parent empowerment by validating their choices and providing them with knowledge and tools to make informed decisions.

### Limitations

Although this study has several strengths, certain limitations should be acknowledged, alongside some recommendations for future research. First, the study included both participants who currently have an excessively crying baby and parents who experienced excessive crying in the past. Perceptions may differ between mothers currently coping with excessive crying and those reflecting retrospectively on a past experience. Nevertheless, both perspectives are highly relevant: the immediate experience offers insight into present challenges, while retrospective accounts provide valuable reflections. Although this approach introduces certain limitations, the diversity of the sample enriches the findings by incorporating unique viewpoints. These varied experiences offer additional insights that might otherwise be overlooked.

Second, there may be sampling bias, as mothers who were dissatisfied with the perceived understanding and support might have been more likely to participate than those without issues. Moreover, this study only involved *mothers* with excessively crying babies. This might be seen as a limitation as fathers, comothers, and same-sex fathers are equally important groups. Fathers experience a lot of distress and have a high chance of developing depression when having an excessively crying baby [12]. Therefore, future studies and interventions need to include other primary caregivers. However, the focus on mothers did ensure a consistent and homogeneous sample, reducing variability in responses that could arise from differences between mothers and fathers or comothers and same-sex fathers.

Third, we have no information about the age of the babies at the time of the excessive crying complaints. This lack of age-specific data is unfortunate, as perceptions of excessive crying and the need for support are likely to vary depending on the infant’s age and developmental stage. Not accounting for this variable may have affected how mothers reflected on their experiences and expressed their needs. However, this broad inclusion strategy was a deliberate choice, as it allowed us to explore general themes that are widely shared across the population. Given the exploratory nature of this study, capturing a broad range of experiences was valuable in identifying common patterns and concerns among mothers.

A fourth limitation is that we did not collect information about the cultural or ethnic backgrounds of the participants. Although the recruitment strategies (eg, Dutch language online communities in Belgium and the Netherlands) suggest that most participants were likely part of a Western European cultural context, we cannot draw firm conclusions about the cultural diversity within the sample. This limits the transferability of the findings to mothers from other cultural backgrounds, whose perceptions and support needs around excessive infant crying may differ. Future research should aim to include and differentiate between diverse cultural perspectives to gain a more inclusive understanding [44,45].

Finally, another potential limitation is related to the measurement instrument. Specifically, the use of “perfect” as the positive anchor on the Likert scale is less common in previous research. Although we deliberately chose to capture participants’ perceptions of an ideal or optimal experience, the word “perfect” may have been perceived as an unattainable ideal and therefore selected less frequently. Future research might consider using a more moderate and relatable term (such as “excellent”) to capture positive evaluations more accurately.

Finally, we did not use any existing/adapted items from validated tools as we believed there did not exist any fitting items in existing tools. However, as a compensation, we used a pilot test (n=3).

In addition to the aforementioned limitations, several final recommendations for future research can be proposed to further advance understanding in this field. For instance, future studies could investigate the association between perceived understanding and support and the specific type of health care professional involved. Given the considerable variation in approaches, treatment methods, and patient interactions across health care disciplines, such an examination would clarify the unique contributions and challenges associated with each. Further, future research might focus on the validation of the conceptual maternal support framework, while considering the previously stated stakeholder groups, so that the maternal support framework can be expanded to the parental support framework.

### Conclusion

This study highlighted the significant challenges faced by mothers of excessively crying infants, emphasizing the critical role of understanding and support from their partners, personal networks, and health care professionals. Findings indicate that health care professionals provide the least understanding and support, followed by personal networks, while partners offer more consistent support and understanding.

Qualitative insights further underscore the emotional and practical support needs of mothers, including a desire for more empathy, respect for their decisions, and tangible support. The maternal support framework developed in this study offers a comprehensive guide to understanding and addressing maternal support forms. It calls for greater collaboration among partners, personal networks, and health care professionals to alleviate the mental and emotional burdens of parents, ultimately improving the well-being of both parents and infants. Future interventions should focus on bridging the gaps in understanding and support, fostering a more supportive environment for families dealing with excessive crying.

## Appendix

Multimedia Appendix 1 Questionnaire.

## Abbreviations

ICPW: Infant Crying and Parent Well-Being
IMH: infant mental health

## Data Availability

The complete codebook, including the fundamental principles (shared support forms) and specific support forms, supplemented with the ad verbatim codes, which stayed close to the original words, will be available upon request. A list of support forms and subforms, each with a verbatim quote, will also be available upon request.
