# Supplementary material for: The Maternal Support Framework Studying Mothers’ Perceived Understanding and Support During Excessive Infant Crying: Exploratory Qualitative Study
Source: JMIR Pediatr Parent. 2025 Oct 28;8:e75669. doi: 10.2196/75669 (PMC12614867; doi:10.2196/75669)
Supplement: Multimedia Appendix 1 [file pediatrics_v8i1e75669_app1.docx]

**Multimedia** **Appendix 1.** Questionnaire.

|  | **QUESTION** | **QUESTION FORMAT** |
| --- | --- | --- |
| *Crying duration* | 1. Indicate **how much your baby cries/cried**…  - Throughout the entire day; - During the morning; - During the afternoon; - During the evening; - During the night. | Multiple choice question |
| *Partner understanding & support* | 1. How do/did you perceive the understanding of your **partner**? | Six-point Likert scale ranging from one (poor/none) to six (perfect) |
|  | 1. How do/did you perceive the support of your **partner**? | Six-point Likert scale ranging from one (poor/none) to six (perfect) |
|  | 1. How do you want to be understood and supported by your **partner**? | Open-ended question |
| *Personal network understanding & support* | 1. How do/did you perceive the understanding of your **personal network** (parents, grandparents, friends, colleagues, etc.)? | Six-point Likert scale ranging from one (poor/none) to six (perfect) |
|  | 1. How do/did you perceive the support of your **personal network** (parents, grandparents, friends, colleagues, etc.)? | Six-point Likert scale ranging from one (poor/none) to six (perfect) |
|  | 1. How do you want to be understood and supported by your **personal network** (parents, grandparents, friends, colleagues, etc.)? | Open-ended question |
| *Health care professional understanding & support* | 1. How do/did you perceive the understanding of the **health care** professionals you consulted? | Six-point Likert scale ranging from one (poor/none) to six (perfect) |
|  | 1. How do/did you perceive the support of the **health care professionals** you consulted? | Six-point Likert scale ranging from one (poor/none) to six (perfect) |
|  | 1. How do you want to be understood and supported by the **health care professionals** you consulted? | Open-ended question |
| *Consulted health care professionals* | 1. **Which health care professionals** did you consult? | Open-ended question |
